# Non-Habit-Related Oral Squamous Cell Carcinoma: A Review

**DOI:** 10.7759/cureus.54594

**Published:** 2024-02-21

**Authors:** Rosalyn Lalremtluangi, Suwarna Dangore-Khasbage

**Affiliations:** 1 Oral Medicine and Radiology, Datta Meghe Institute of Higher Education & Research, Wardha, IND

**Keywords:** immunosuppressive, prognosis, oropharyngeal, carcinoma, biomarkers

## Abstract

Oral squamous cell carcinoma (OSCC) is a serious and potentially life-threatening condition that can have a profound impact on an individual’s health and well-being. Its etiology is commonly known to be habit induced, such as tobacco consumption, smoking, or alcohol abuse. Apart from these etiologies, certain factors that lead to OSCC are also present but are less frequently encountered in hospitals and clinics. However, these non-habitual factors, with their pathogenesis, can lead to OSCC, which may be confusing to certain medical practitioners. This article discusses the various non-habitual causes that can lead to OSCC, as well as their pathophysiology, molecular expression, and related indicators and prognostic factors.

## Introduction and background

Oral squamous cell carcinoma (OSCC) is the most common variety of malignancies in the head and neck region [1,2], and it poses a serious threat to the life quality of people who are suffering from it. According to data obtained from the Global Cancer Observatory, the annual incidence of OSCC in 2020 was 377,713 cases worldwide, with Asia being the highest among other continents [3,4]. Habits such as tobacco use, alcohol intake, and smoking are the leading causes of OSCC, accounting for at least 80% of cases [5]. Tobacco contains numerous harmful substances, which may include nitrosamine, acrolein, carbon monoxide, formaldehyde, and polonium-210, all of which, through chronic irritation and inflammation of the oral mucosa, may cause the formation of premalignant lesions that may eventually lead to oral malignancy [6]. Both drinking and smoking are independently and synergistically linked to an increased risk of developing oral cancer, and the risks typically rise with greater exposure. Despite the growing awareness about the benefits of quitting tobacco, smoking, and drinking, the number of occurrences of oral malignancies has not declined much [7].

Nevertheless, OSCC, which occurs without any deleterious oral habits, has also been reported in the literature. According to recent research in 2019 by Saxena et al., 4-6% of oral cancer incidences are not associated with any oral habits in the Indian subcontinent [8]. Several etiologic factors may be present that cause OSCC without any oral habits. Age and gender predilection have been reported, along with conditions like hereditary and genetics, food habits, ultraviolet (UV) light, human papillomavirus (HPV) infection (where evidence of an elevated HPV DNA positive of 20-64% in oral lesions could be premalignant), and immune suppression, which are believed to be the non-habitual causes of the lesion [8,9]. The bulk of studies in past years did not make it quite clear as to which factors may trigger or cause OSCC in a non-habitual patient. As a result, this article aims to review the various non-habitual factors that can potentially lead to OSCC, along with their pathogenesis, markers, molecular expression, and prognostic aspects.

## Review

Non-habitual factors related to OSCC

Age

Regarding the age group that is most frequently impacted by non-habit-related OSCC, there are conflicting statistics available [10]. While some writers claim that non-habit-related OSCC frequently affects young people, others claim that the age of susceptibility is bimodal globally, i.e., between 40 and 70. However, this situation deviates from the bimodal age predilection [11]. The earlier age at which non-habit-associated OSCC develops is thought to be influenced by genetic predisposition, viral etiology, phagocytic diseases, Fanconi anemia (FA), and environmental toxins [11]. The cumulative effect of dietary and environmental carcinogens is thought to make older people more prone to cancer [12]. Given the patient’s advanced age, frequent trauma in this situation could act as a mutagen, kicking off carcinogenesis in a favorable oral environment [13].

Gender

For oral cancer without a history of habits, women are more likely to develop it worldwide as compared to men. Male predilection is more associated with habit-induced OSCC [14]. It is well known that cancer cell lines originating from head and throat malignancies have genes involved in the metabolism of estrogen, especially estradiol, in tongue cancer. In females with non-habit-related OSCC, other research has linked altered levels of luteinizing hormone, follicle-stimulating hormone, prolactin, and testosterone [14]. This case widens the gaps in knowledge regarding the precise function of estrogen receptors in non-habit-associated OSCC, which appears to favor females [15].

Hereditary

Despite the fact that OSCCs are rare in communities, epidemiological research has indicated that there may be inherited risks for OSCCs. Studies have shown that the autosomal dominant mode of inheritance is seen in 0.94% of total oral cancer in India. Nevertheless, little is known about the genetic predispositions that lead to OSCCs. VAV2 and IQGAP1 were found to be the genetic causes of an oral cancer case in a family by Huang et al. In the family with oral cancer, both genes showed heterozygous mutations and were inherited autosomal dominantly [16]. According to a case study by Jeong et al., there is a little girl with OSCC whose mother had OSCC in her family history. It was discovered that the patient’s mother also carried the germline mutation CDKN2A c.301G>T [17]. As a result, germline mutations may be very important in the development of OSCC. It is thought that the rare hereditary condition known as FA increases the risk of head and neck squamous cell carcinomas by 200-800 times [18]. The incapacity of FA to heal DNA interstrand cross-links is one of its defining characteristics. Treatment for OSCC in FA is challenging since patients are not tolerant of the available treatments, which include platinum-based chemotherapy and ionizing radiation. In FA, the majority of tumors are found at an advanced stage [19].

Nutrition and Diet

Foods may directly cause cancer through the carcinogens they contain, or they may indirectly cause cancer through the metabolism’s production of carcinogens. de Oliveira Bezerra et al. state that 11-15% of oral and pharyngeal malignancies globally are caused by disequilibrium and/or a food deficit [20,21]. The majority of individuals eat a wide range of foods that provide a variety of nutrients, and their dietary habits frequently alter throughout time in response to cultural and socioeconomic factors [21]. An eating pattern that includes a lot of animal-based foods, fats, sugars, processed foods, and relatively little complex carbs and fiber has gradually replaced “traditional” eating patterns based on the consumption of grains and cereals due to the so-called “nutritional transition” [22]. These eating habits are thought to be potentially beneficial for the development of oropharyngeal cancer. Consuming animal fat-rich foods (dairy products, processed meats, and a variety of meats) and high-fat sauces has been positively linked to an elevated risk of OSCC [23]. An increased risk of mouth cancer has also been linked to a low fruit and vegetable diet. Certain vitamins, like vitamin E, have been linked to a decreased risk of cancer because they include substances like tocopherol, which has anti-inflammatory and antioxidant properties. However, there is no evidence that the vitamins B1, B5, B6, and K can prevent oral cancer [23].

Physical Carcinogens

UV exposure to sunlight is a potent etiological and predisposing factor for OSCC in a non-habitual patient. Jobs that include working outside and being in the sun for extended periods of time are more likely to cause OSCC on the lips. The incidence of OSCC due to UV radiation is estimated to be 3.33 or 3.94 per 100,000 individuals worldwide [24]. Effective defense systems have been developed to shield epidermal keratinocytes from UV-induced tumor development. These rely on signaling pathways that cause apoptosis to be induced [25]. However, because of the deregulation of these pathways, SCC cells have acquired certain traits [26]. UV light can cause inhibition of the apoptosis process, and as a result, the apoptosis deficit seems more important than increased proliferation overall. The most important causes of inhibition of apoptosis may be COX-2 activation, reactive oxygen species production, and p53 inactivation [27].

Ionizing radiation is also known to cause mutagenesis, which may include X-rays, beta, alpha, and gamma radiation, as well as radioactive isotopes. There are several studies regarding X-rays that indicate an increased chance of developing oral cancer due to their exposure [28]. Ionizing radiation is potent enough to break free electrons from atoms that are firmly bonded, damaging DNA and perhaps causing mutations that aid in the development of cancer. Dental X-rays are a common source of exposure to ionizing radiation in the oral cavity. Oral cancer risk may rise with frequent or high-dose exposure to ionizing radiation, particularly in medical settings, even if the doses from routine dental X-rays are generally thought to be modest and the risk is minimal [28,29]. The diagnostic benefits of X-rays, including the detection of dental issues and oral or other medical conditions, are often considered to outweigh the potential risks that can cause radiation exposure [29]. Healthcare professionals adhere to standard protocol to ensure that radiation exposure is maintained as low as reasonably attainable in order to minimize the risk of radiation-induced mouth cancer. This entails employing thyroid collars and lead aprons to protect non-imageable regions and utilizing the lowest feasible radiation dosage for medical evaluations [30].

Biological Carcinogens

HPV has been linked to a number of malignancies in humans, including those in the head and neck, vulvar, vaginal, penile, and cervical regions. The incidence of OSCC with a positive HPV infection has risen from less than 20% to more than 70% globally. Low-risk HPVs, such as HPV6 and HPV11, cause benign warts, while high-risk HPVs, such as HPV16 and HPV18, cause premalignant squamous intraepithelial neoplasia that can progress to malignant lesions. It is unclear how OSCC related to HPV behaves clinically [31]. For men under 50, the incidence of OSCC linked to HPV has sharply increased. A recent report given by Mirghani et al. showed a 17.5% positivity for high-risk HPVs in oral epithelial dysplasias using in situ hybridization and immunohistochemical staining against p16INK4A, a hallmark of infection by a biologically active virus [32]. People who have oral cancer caused by HPV are less likely to smoke or drink, and their prognosis is usually favorable. The floor of the mouth (9-42%) and the tongue (8-25%) appear to be the most common areas of HPV infection, despite frequency variations across research [33,34].

Epstein-Barr virus (EBV), which is a member of the herpesvirus family, has also been linked to the development of certain malignancies, including lymphomas and nasopharyngeal carcinoma [35]. In contrast to its participation in other malignancies, the correlation between EBV and OSCC is less evident. Research has suggested a potential connection between EBV and oral cancer, particularly in specific subsets of patients. While the precise methods by which EBV may aid in the development of oral cancer remain incompletely known, several investigations have found EBV in the tissues of oral cancer patients, indicating that the virus may be involved in the initiation or advancement of specific cases of mouth cancer [36]. It may contribute to oral malignancy by promoting genetic instability or affecting immune responses. The malignancy of EBV is associated with a number of proteins found in the viruses that regulate immune response, cell apoptosis, and cell proliferation [37]. Latent membrane proteins help in activating the signaling pathway, whereas the nuclear antigens of EBV help in gene expression. The impact of EBV on oral mucosal carcinogenesis, however, is still unknown because different methods, such as PCR, nested PCR, RT-qPCR, IHC, and ISH, are used to detect the virus [37]. The sensitivity and specificity of these tests vary depending on the method employed, which results in varying relationships between EBV infection and OSCC [38].

Oral Lichen Planus (OLP)

OLP is a chronic inflammatory T cell-mediated disease that mostly affects the tongue and buccal mucosa. Although the etiology is clearly unknown, T lymphocyte autoreaction may be of primary importance for the progression of OLP. These cells cannot differentiate between foreign antigens and inherent molecules of the body [39]. Stress, systemic medications such as beta-blockers, nonsteroidal anti-inflammatory drugs, antimalarials, diuretics, oral hypoglycemics, and penicillamine, oral retroviral medications, hypersensitivity to dental materials, chronic liver disease and hepatitis C virus, and graft-versus-host disease are also believed to cause OLP [40]. Over the past years, it has been clear that the immune system plays a major part in the onset of this illness. The histopathologic features of a subepithelial infiltration dominated by T lymphocytes and macrophages, as well as the degeneration of basal cells known as liquefaction degeneration, corroborate this [40]. These characteristics can be understood as a manifestation of the immune system’s cell-mediated component contributing to the etiology of OLP by means of T-lymphocyte cytotoxicity targeted at antigens produced by the basal cell layer. Clinical symptoms may include red and white elements, together with different textures and clinical types, which may include reticular, papular, plaque-like, bullous, erythematous, and ulcerative [41].

The foremost incidence of OLP malignant transformation was reported in 1910. A recent meta-analysis reported that 1.1% of lesions of OLP evolve into OSCC worldwide, where a higher frequency among hepatitis C virus-positive individuals was seen [41]. Tampa et al.’s research reveals a number of biomarkers that, when present, induce OLP to proceed to OSCC. These include variables that modulate apoptosis (p53 and MCL-1), cell cycle regulators (BMI1 and p16), tissue remodeling factors (MMPs), and factors related to inflammation (TNF-α, IL-6, and COX-2) [39]. The type of OLP that appears to change into OSCC most frequently is erosive and erythematous OLP. This could be due to the lack of protective mechanisms in the mucosa in erosive and erythematous OLP, unlike other types of OLP where there is keratinization as a defense reaction to stimulus. This lack of a defense system in erosive and erythematous OLP could lead to more rapid and aggressive subepithelial changes as compared to other types, which eventually could cause oral malignancy [42,43]. Malignant transformation most frequently happens in localized tongue lesions. According to Andabak-Rogulj et al., OLP lesions require an average of five and a half years to evolve into an established OSCC [44].

Immune System Suppression

Tumor cells have the ability to create a variety of immunosuppressive chemicals, such as regulatory T cells, tumor-associated macrophages, tumor-associated neutrophils, and cancer-associated fibroblasts [45]. These substances can impede the immune cells’ natural ability to fight cancer. When compared to non-immunosuppressed patients, immunosuppression has been shown to approximately double the risk of cancer-specific outcomes (such as recurrence and overall survival). Additionally, immunosuppression has a substantial correlation with poor outcomes in OSCC, with a risk of 0.2-1% [46]. Tumor cells can evade immune surveillance and tumor immunity through immunosuppressive mechanisms facilitated by their contact with immune cells. This ultimately advances the cancerous process. There was a significant correlation observed between the prognosis of OSCC in immunosuppressed patients and 11 immunosuppressive genes: CXCL8, TLR3, IL22, ORMDL3, FGFR3, CTLA4, HPRT1, BGLAP, CALCA, SPHK1, and INHBB [47]. Two survival subtypes, one with a lower chance of survival and the other with a higher probability of survival, were distinguished between OSCC patients using a deep learning-based model. A number of signaling pathways implicated in immunosuppression (such as T cell and B cell receptor signaling, p53, Notch, JAK-STAT, and MAPK) that are enriched in the aggressive subtype of OSCC have been identified as potential therapeutic targets. These pathways may be useful in treating immunosuppressed patients with OSCC and enhancing overall survival in particular patient populations [48].

Trauma

Apart from the oral habits that are commonly known to cause OSCC, an increased risk of oral cancer may be attributed to chronic inflammation due to poor dental hygiene, sharp edges from damaged teeth, or prolonged irritation from stimulation such as ill-fitting dentures. A report given by Pentenero et al. revealed that 44% of OSCC on the tongue showed signs of mechanical trauma. The risk of oral malignancy may also be higher in people who participate in practices that cause repeated stress to the oral cavity, such as biting the oral mucosa on a regular basis [49].

When in direct interaction with an agent that is mechanical, such as teeth or a denture, during functional or parafunctional movements or in the decubitus posture, the presence of chronic and persistent mechanical irritation can result in erythema, atrophy, ulceration, keratosis, hyperplasia, indentation, or fibrosis. Since most of the mucosal surfaces in the oral cavity have physiological contact with teeth or dentures when moving functionally or when at rest, it is easy to see that there is the necessary straight contact between the lesion and the traumatic agent. This is especially true when OSCC is present, which frequently indicates altered surfaces of mucosa because of an increased volume [50]. Through mechanisms that involve the interruption of the extracellular matrix’s normal architecture, which promotes expression of oncogenes, status of hyperproliferation, favorable inflammatory microenvironment, and enabled exposure to carcinogens, a potential protagonist for chronic trauma in the process of carcinogenesis has been suggested [51].

## Conclusions

OSCC is a common malignancy in the head and neck region, usually caused by deleterious habits. However, apart from these habits, several non-habit-induced OSCCs are also present, which have not been thoroughly recognized in society. Understanding the multifaceted nature of non-habit-induced OSCC requires a holistic approach that considers genetic, viral, immunological, environmental, hormonal, and psychosocial factors. Also, OSCC could be prevalent in certain people with occupations that require long-span exposure to sunlight. Chronic irritation from traumatizing objects such as improperly fitted dentures, impinging dental restorations, pointed cusps, and root fragments can also cause damage to the oral cavity, potentially leading to OSCC. This reveals the challenging aspect of oral cancer that requires ongoing investigation and a multidisciplinary approach to enhance the ability to prevent, diagnose, and treat this condition effectively in patients without oral habits. Collaborative efforts between clinicians, researchers, and public health initiatives are essential. Through continued research and partnership, the medical community can strive toward a more comprehensive understanding of non-habit-induced OSCC and ultimately improve outcomes for affected individuals.
